# Potential distributions of *Bacillus anthracis* and *Bacillus cereus* biovar *anthracis* causing anthrax in Africa

**DOI:** 10.1371/journal.pntd.0008131

**Published:** 2020-03-09

**Authors:** Daniel Romero-Alvarez, A. Townsend Peterson, Johanna S. Salzer, Claudia Pittiglio, Sean Shadomy, Rita Traxler, Antonio R. Vieira, William A. Bower, Henry Walke, Lindsay P. Campbell

**Affiliations:** 1 Department of Ecology & Evolutionary Biology and Biodiversity Institute, University of Kansas, Lawrence, Kansas, United States of America; 2 Bacterial Special Pathogens Branch, Division of High-Consequence Pathogens and Pathology, National Center for Emerging and Zoonotic Infectious Diseases, Centers for Disease Control and Prevention, Atlanta, Georgia, United States of America; 3 Food and Agriculture Organization of the United Nations, Animal Health Service, Animal Production and Health Division, Rome, Italy; 4 One Health Office, National Center for Emerging and Zoonotic Infectious Diseases, Centers for Disease Control and Prevention, Atlanta, Georgia, United States of America; 5 Florida Medical Entomology Laboratory, Department of Entomology and Nematology, IFAS | University of Florida, Vero Beach, Florida, United States of America; Beijing Institute of Microbiology and Epidemiology, CHINA

## Abstract

**Background:**

*Bacillus cereus* biovar *anthracis* (Bcbva) is an emergent bacterium closely related to *Bacillus anthracis*, the etiological agent of anthrax. The latter has a worldwide distribution and usually causes infectious disease in mammals associated with savanna ecosystems. Bcbva was identified in humid tropical forests of Côte d’Ivoire in 2001. Here, we characterize the potential geographic distributions of Bcbva in West Africa and *B*. *anthracis* in sub-Saharan Africa using an ecological niche modeling approach.

**Methodology/Principal findings:**

Georeferenced occurrence data for *B*. *anthracis* and Bcbva were obtained from public data repositories and the scientific literature. Combinations of temperature, humidity, vegetation greenness, and soils values served as environmental variables in model calibrations. To predict the potential distribution of suitable environments for each pathogen across the study region, parameter values derived from the median of 10 replicates of the best-performing model for each pathogen were used. We found suitable environments predicted for *B*. *anthracis* across areas of confirmed and suspected anthrax activity in sub-Saharan Africa, including an east-west corridor from Ethiopia to Sierra Leone in the Sahel region and multiple areas in eastern, central, and southern Africa. The study area for Bcbva was restricted to West and Central Africa to reflect areas that have likely been accessible to Bcbva by dispersal. Model predicted values indicated potential suitable environments within humid forested environments. Background similarity tests in geographic space indicated statistical support to reject the null hypothesis of similarity when comparing environments associated with *B*. *anthracis* to those of Bcbva and when comparing humidity values and soils values individually. We failed to reject the null hypothesis of similarity when comparing environments associated with Bcbva to those of *B*. *anthracis*, suggesting that additional investigation is needed to provide a more robust characterization of the Bcbva niche.

**Conclusions/Significance:**

This study represents the first time that the environmental and geographic distribution of Bcbva has been mapped. We document likely differences in ecological niche—and consequently in geographic distribution—between Bcbva and typical *B*. *anthracis*, and areas of possible co-occurrence between the two. We provide information crucial to guiding and improving monitoring efforts focused on these pathogens.

## Author summary

*Bacillus cereus* biovar *anthracis* (Bcbva) is an emergent pathogen causing anthrax in West and Central African countries. It shares multiple bacteriological and genomic characteristics with *Bacillus anthracis*, the gram-positive bacterium causing anthrax in mammals worldwide. However, Bcbva has only been isolated in tropical humid forested environments in Africa; thus, we attempted to characterize its potential distribution and the differences with *B*. *anthracis* using an ecological niche modeling framework. According to our results, both pathogens are using different environments and their distribution looks complementary with *B*. *anthracis* occupying savanna-like environments and Bcbva occupying forested areas; further, we detected some level of overlap between the potential geographic distributions of *B*. *anthracis* and Bcbva, despite their lack of overlap in their current environmental space. This is the first time the potential distribution of Bcbva is addressed considering all the available spatial information on the pathogen occurrence available as of 2017. Although our Bcbva models are limited due to sample size, this exploration informs on areas that would likely be considered for further investigation of Bcbva outbreaks with special emphasis on areas where Bcbva and *B*. *anthracis* could co-exist.

## Introduction

*Bacillus anthracis*, the causative agent of anthrax, affects humans, livestock, and wildlife in multiple regions around the world. It is a gram-positive, rod-shaped, and spore-forming bacterium that primarily affects wild and domestic herbivores, resulting in high mortality rates, while also posing an important human health risk [1]. The primary route of infection with anthrax in wildlife and livestock is grazing in vegetated areas where previous anthrax outbreaks have occurred [2,3]. Spores residing in soils, vegetation, or water are ingested, resulting in germination, followed by rapid replication of vegetative cells and elaboration of exotoxins, leading to septicemia and death [4]. Carcasses opened by scavenger animals or via human activities during disposal exposes vegetative cells to oxygen, which results in spore formation within 48–72 hours (but see [5]). Additional transmission routes in animals include mechanical from biting hemophagic flies [6–9], in addition to a possible respiratory pathway through spore inhalation, although just documented in one incident [10]. In humans, cutaneous anthrax is the most common clinical outcome following contact with contaminated animal hides or animal products [1]. Consumption of meat from infected animals can lead to gastrointestinal anthrax, which although rare compared to cutaneous anthrax, continues to cause problems in many developing regions of the world [1,11]. Inhalation anthrax in humans presents with severe respiratory symptoms, accompanied by high mortality rates, and poses an important bioterrorism risk, as was demonstrated during the 2001 anthrax attacks in the United States [12]. Anthrax through injection is the least common form of the disease in humans, and was described as a clinical entity affecting mainly heroin users [13].

The worldwide distribution of *B*. *anthracis* is a characteristic derived directly from its ability to form spores that are resistant to diverse environmental conditions [1,4,7,14,15]. The ability of spores to persist for years and even decades in the environment is well-documented, including several reports of spore survival lasting >50 years [1,4,7,16–18]. Mounting evidence suggests an association between *B*. *anthracis* spore survival and elevated soil pH values, high calcium concentrations, and presence of organic matter in the natural environment, although direct links between spore decay and specific environmental variables remain under investigation [1,3,4,14].

In sub-Saharan Africa, anthrax occurs in wildlife and domestic livestock in multiple countries, with periodic, scattered epizootic outbreaks [19]. Most outbreaks occur in savanna ecosystems, where extensive livestock grazing and animal husbandry takes place [1]. In addition to agricultural livestock, these regions hold many wildlife preserves populated by susceptible ungulate species, such as African buffalo (*Syncerus caffer*), greater kudu (*Tragelaphus strepsiceros*), hippopotamus (*Hippopotamus amphibius*), zebra (*Equus quagga*), and elephant (*Loxodonta africana*), among others [7,16].

After the discovery of a series of chimpanzee (*Pan troglodytes troglodytes*) and gorilla (*Gorilla gorilla gorilla*) carcasses in 2001 and 2004–2005 in Côte d’Ivoire and Cameroon [20,21], *B*. *anthracis* was incriminated as a new emerging infectious disease hazard within forested environments in sub-Saharan Africa, including yet another risk to endangered species such as great apes [22]. However, further detailed molecular, morphological, and microbiological analyses revealed that the pathogen was in fact a new variety of a different *Bacillus* species, *Bacillus cereus* biovar *anthracis* [23,24] (henceforth “Bcbva” for brevity). Phylogenetic analyses determined that *B*. *anthracis* and Bcbva fall within the broader *B*. *cereus* group, comprising multiple species with diverse pathogenicity and ecological niches [25]. Bcbva exhibits chromosomal characteristics associated with *B*. *cereus*, but contains two virulence plasmids almost identical to those in *B*. *anthracis* [24]. Experimental studies in mice and guinea pigs demonstrated Bcbva virulence comparable to that of wild-type *B*. *anthracis*; similarly, protection against Bcbva infection was conferred using vaccination with formaldehyde-inactivated *B*. *anthracis* spores plus protective antigen [26].

Further studies have isolated Bcbva from animal anthrax cases in West and Central African countries, including Liberia, Democratic Republic of Congo, and Central African Republic [27,28]. Importantly, Bcbva was found to infect a wide range of species, including not only gorillas and chimpanzees, but six additional monkey species, duikers, mongooses, and porcupines; the pathogen was responsible for high wildlife mortality rates in forested areas within Tai National Park [28,29]. Recent mathematical models have shown considerable potential of Bcbva to undermine chimpanzee populations [28]; considering its similarities to *B*. *anthracis*, the U.S. Department of Health and Human Services added Bcbva to its list of select agents and toxins, along with viruses such as Ebola and Marburg [30].

Here, we focus on characterizing the ecological niches of *B*. *anthracis* and Bcbva in sub-Saharan Africa and on evaluating potential niche differences between the two pathogens. The potential distribution of *B*. *anthracis* links closely to locations of previous anthrax outbreaks, but understanding of environmental factors leading to emergence and risk across geographic regions remains incomplete. Although *B*. *anthracis* has been documented from multiple continents and is considered to have a global distribution [15,17], Bcbva infections have been identified only within sub-Saharan Africa [27,28]. Empirical evidence suggests that Bcbva infections occur in subtropical humid environments, primarily in non-human primates, whereas *B*. *anthracis* infections occur more commonly in arid regions, and so far, the pathogen has been found in a broader variety of host species, although susceptibility varies among them; identification of additional Bcbva host species will likely increase with further investigations [7,8,16]. Although locality data for Bcbva are sparse in comparison to those for *B*. *anthracis*, these observations suggest that Bcbva may occupy an ecological niche peripheral to that of *B*. *anthracis*, which may explain high incidence in humid, forested environments.

Recent advances in geospatial analysis approaches provide new opportunities to explore and predict the geographic distribution of suitable environments under which potential spore survival and possible anthrax transmission may occur [31]. Ecological niche modeling is a correlative approach that uses georeferenced occurrence data and environmental variables to identify the geographic distribution of environments suitable for a species of interest [32]. This approach has been used to anticipate potential distributions of multiple pathogens at global and regional scales [33–35], including previous anthrax investigations [36–42].

The objective of this study is to take a broad-scale approach to understanding the potential geographic distributions of *B*. *anthracis* and Bcbva in sub-Saharan Africa, using an ecological niche modeling approach. A second objective is to quantify the similarity of environments from which Bcbva isolates have been obtained with those occupied by *B*. *anthracis* in West Africa. Understanding potential niche overlap or difference between these pathogens will provide new insights into possibilities for co-occurrence, or for infection from Bcbva in previously unknown geographic areas. This information is critical to identify areas to build veterinary and public health capacity to properly differentiate anthrax outbreaks caused by either *B*. *anthracis* or Bcbva in Africa.

## Methods

### Occurrences

Confirmed occurrence records for anthrax cases in animals or humans from Africa were compiled from multiple sources, including publicly available data from the Emergency Prevention System (EMPRES) of the Food and Agriculture Organization (FAO) of the United Nations [17] and records documented by the online outbreak databases HealthMap [43] and ProMED-mail [44–46]. We also manually georeferenced localities from [47–50]. For Bcbva occurrences, we used all confirmed localities available for the pathogen until December 2017 as reported by [21,23,24,27,28]. We used exact coordinates when reported [27,28], or georeferenced them manually according to the information provided [21]. All occurrence data are provided in S1 Table.

**Fig 1:**
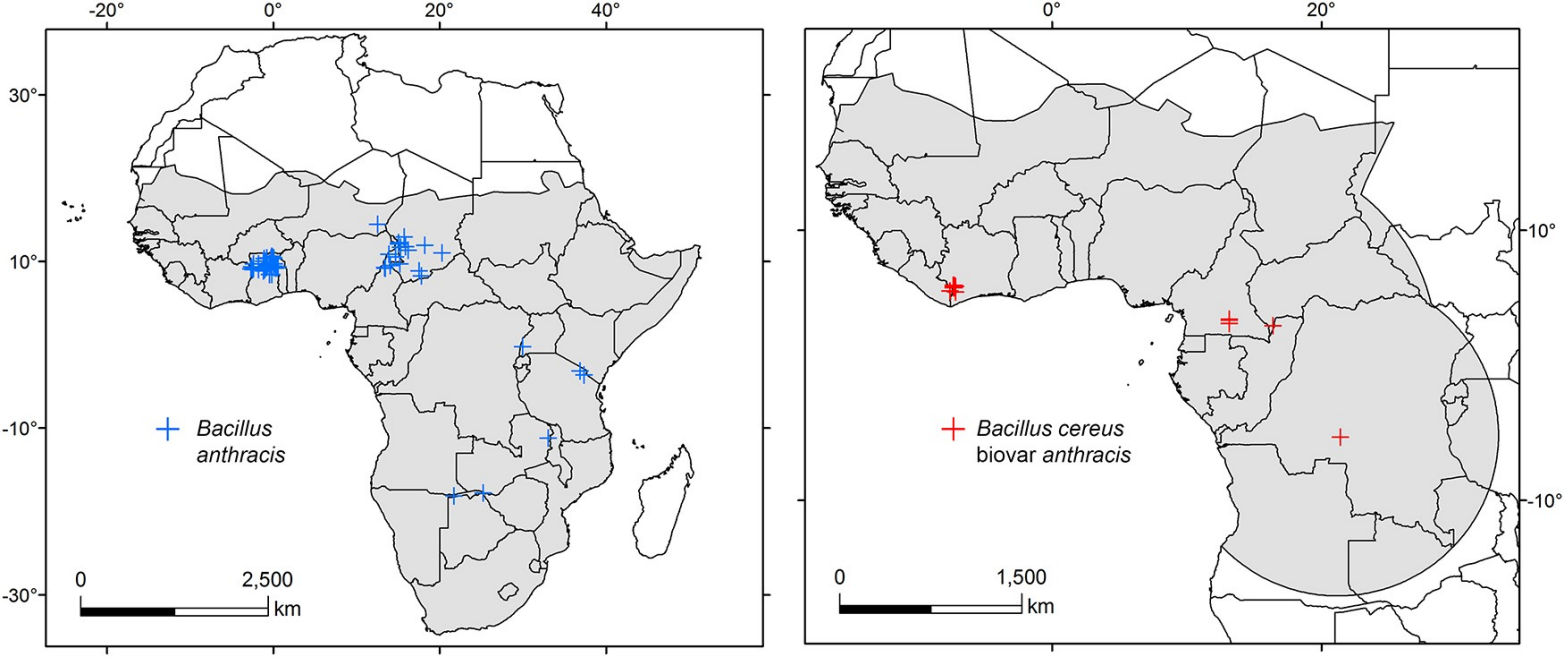
Occurrence points and calibration areas. Confirmed records of *Bacillus anthracis* (blue) and Bcvba (red) with their corresponding calibration areas (gray shading). Maps were developed using shape files of Africa from the public domain repository of Natural Earth (http://www.naturalearthdata.com/) and built with ArcGIS 10.3 (ESRI Redlands, CA, USA).

We accepted polymerase chain reaction (PCR) and sequencing, and bacteriological examination as reasonably certain laboratory confirmation for *B*. *anthracis*, except for Ghana where we included georeferenced confirmed outbreaks [47,48]. We also removed duplicate records, records with locational information only referring to centroids of first and second political administrative levels (e.g., centroid of the country, state, or province), and filtered occurrences to retain only one per grid cell. Many African countries have reported suspected occurrences of *B*. *anthracis*; however, confirmed anthrax records were obtained only from Bostwana, Cameroon, Chad, Ghana, Namibia, Tanzania, Uganda, and Zambia (Fig 1 and S1 Table). Occurrence data for Bcbva were obtained from Cameroon, Central African Republic, Côte d’Ivoire, Democratic Republic of the Congo, and Liberia (Fig 1 and S1 Table).

Occurrence dates ranged from 2000 to 2017. We obtained a total of 57 occurrences for *B*. *anthracis*, and 14 for Bcbva. To prevent overfitting owing to spatial autocorrelation and sampling bias (e.g., occurrences in Ghana, Fig 1), we thinned our data spatially using a distance of 30 km (*B*. *anthracis* n = 40, Bcbva n = 7) and 50 km (*B*. *anthracis* n = 34, Bcbva n = 5) and developed models using all occurrences in each thinned dataset [51].

### Calibration areas

Ecological niche models are sensitive to the area selected for calibration, and evaluation metrics can be inflated as an artifact of including larger study areas [52]. We selected specific calibration areas for each pathogen considering its natural history. As mentioned, *B*. *anthracis* has a nearly global distribution [1,15,17]; its spore form can endure a broad range of environmental conditions although the capacity for spore survival declines in extreme settings [7]. For this reason, we defined all of sub-Saharan Africa as the calibration area for *B*. *anthracis* [53]. For Bcbva, we wanted to use an area broad enough to include areas surrounding its known occurrences [23]. We developed a buffer of 12° (~1300 km) around available occurrences including areas south of the Sahara Desert across western and central Africa (Fig 1). In this context, the two calibration areas (Fig 1) represent hypothesis of the geographic regions that the species have been able to explore and potentially colonize through time, which corresponds to the **M** region described by [54].

### Environmental variables

We explored four classes of environmental variables for model calibration: temperature, humidity, vegetation greenness, and soils. All variables were resampled to 5’ (~10 km) spatial resolution to address uncertainty of georeferencing some localities [31]. Climate variables were obtained from the MERRAclim 2000–2010 dataset, a global climatic repository derived from remotely-sensed satellite data and ground information [55]. MERRAclim has advantages over other climate data sets used commonly in ecological niche modeling applications because satellite information helps to reduce uncertainty in interpolated values [55]. We used the so-called “bioclimatic” variables, including temperature and moisture (i.e., humidity) related layers (S2 Table) from the decade 2000–2010 to match the time of georeferenced cases.

Normalized Difference Vegetation Index (NDVI) values (version six, V6) were drawn from biweekly (16-day composite) images at 500 m resolution from the Moderate Resolution Imaging Spectroradiometer (MODIS) sensor on board of NASA’s Terra satellite [56]. NDVI characterizes the amount of green biomass in an area, and has been used as a proxy for soil moisture [57]. We obtained satellite data from 2005 to 2017 for a total of 299 satellite images (available at https://lpdaac.usgs.gov/data_access/data_pool, S2 Table). Original images were downloaded and reprojected to geographic coordinates (WGS 84) using the MODIS Reprojection Tool [58] and the ‘rts’ package implemented in R version 1.0–47 [59,60].

Additionally, we investigated soil variables, including pH, cation exchange capacity (e.g., calcium), and carbon content (S2 Table) from the SoilGrids dataset at 250 m resolution and at multiple depths ranging from 0–5 cm and 5–15 cm [61] available at https://soilgrids.org/. SoilGrids is a global repository of chemical and physical soil properties built by the International Soil Reference Information Centre (ISRIC)—World Soil Information, that includes information for regions with gaps in availability of continuous soils values, including areas across the continent of Africa [61].

To prevent development of overly complex models owing to high correlations among environmental variables in each class of data, we used a separate principal components analysis (PCA) for each set of environments [31,62]. We retained the first three principal components (PCs) for each environmental realm, explaining 99.98% of the variance for temperature, 99.99% for humidity, 91.49% for NDVI, and 97.43% of the variance for soil-related variables; thus, analyses were performed with 12 environmental layers.

### Ecological niche models

We chose a maximum entropy approach implemented in the software package Maxent (version 3.3.3k) [63]. We explored different parameters for our models, considering different combinations of response types (i.e., linear [L], linear+quadratic [LQ], linear+quadratic+product [LQP], and linear+quadratic+product+threshold+hinge [LQPTH]), and regularization multiplier [RM] (i.e., 0.1 to 2, by increments of 0.1, and also RM of 3, 5, 7 and 10) within the Maxent modeling framework for the three sets of occurrence data (i.e., no thinning, thinned to 30 km, and thinned to 50 km) and the environmental variables described (i.e., 12 PCs) [31,64,65]; regarding selection of environmental variables, we noted Maxent’s ability to assign zero-value lambda coefficients (variable weights in models) and included all 12 PCAs. An expanded discussion on our model selection approach can be found in the S1 File.

### Model evaluation

Models for *B*. *anthracis* were generated using a random sample of 50% of occurrence data for calibration and the remaining 50% for model testing [31,64,65]. We used a three-step framework to evaluate and select model outputs recently automated in the kuenm package in R (https://github.com/marlonecobos/kuenm) [66]. First, we assessed statistical significance using the partial area under curve of the Receiver Operating Characteristic (pROC; [67]). Statistically significant models were assessed using omission rates to select those with the best performance using a threshold of 5% training omission rate [32,64]. Finally, models selected through both metrics were further discriminated using the Akaike information criterion corrected for sample size (AICc) to obtain models with low complexity and good fit to the underlying data [64,68,69].

For Bcbva, owing to small sample size, we used a leave-one-out approach [70] developed for such situations (e.g., less than 25 points) [71,72] to assess statistical significance of models. We calculated *p*-values for each model through the software PvalueCompute.exe provided in [70]. Then, we used minimum training presence (MTP) omission rates obtained with the ENMeval package in R [64] to threshold models; independent subsets of occurrence data were overlaid on these binary predictions to calculate omission error as a measure of performance. We calculated AICc values for all Bcbva models except those using 50 km thinned occurrences, for which the number of parameters was higher than the number of available occurrences [68,69]. An expanded discussion on each metric and the current approach can be found in the S1 File.

For pROC and omission rate evaluation metrics, Maxent outputs were assessed in logistic format while for AICc, outputs were based on raw outputs [63,64,73]. All raster data manipulation and evaluations were performed using ArcGIS 10.3 (ESRI, Redlands, CA) and R software (https://www.r-project.org/). Packages used for raster manipulation, model calibration, model evaluations, etc are listed in the S1 File.

### Final models

Parameters identified as the best for each combination of occurrence data (i.e., no thinning, thinned to 30 km, and thinned to 50 km) and environmental variables, were used to develop a final model set for *B*. *anthracis* and Bcbva. We used the selected parameters and Maxent’s logistic output (i.e., a continuous scale from 0 as non-suitable to 1 suitable prediction) in a bootstrap with ten replicates to calculate the median as a representation of suitability for each species; we used the difference between maximum and minimum values (i.e., range) in each pixel as a representation of model uncertainty [32,34] for each case. Final model outputs were classified into binary suitable and non-suitable categories, based on MTP, and an adjusted MTP in which we sought the highest threshold that included 95% and 90% of calibration points (i.e., *E* = 5%, *E* = 10%, respectively, [67]), among all the modeling sets for *B*. *anthracis* [67]. Given the limited number of records available for Bcbva, we used MTP and *E* = 5% as thresholds [67]. Model interpretation for final Bcbva models was constrained to its calibration area to avoid extrapolation of final model parameters to the rest of the African continent.

### Niche similarity

We used a background similarity test to assess possible differences between the potential niches of *B*. *anthracis* and Bcbva and their associated environmental profiles (i.e., temperature, humidity, soils, and NDVI separately, and all together) [74,75]. We used a one-tailed test based on Schoener’s *D* values to compare the observed overlap between the niche models for the two species to a null distribution; the latter was generated using the occurrence points of one of the species, with random models generated from the environmental background of the other species and vice versa; we rejected the null hypothesis of niche similarity if the empiric overlap fell in the lower 5% of the null distribution [74]. Additionally, we used the first two PCs of all environments and the first two PCs of each environmental dimension for *B*. *anthracis* and Bcbva to develop a background similarity test in environmental space, using a scale-free kernel density approach across a gradient of environments as implemented by [76]. All comparisons were performed in R software using the ENMTools R-package available at https://github.com/danlwarren/ENMTools. Calculation of *p*-values was done following the methodology of [77] for one-tailed permutation tests.

## Results

Our modeling approach with three sets of occurrences (i.e., no thinning, thinned to 30 km, and thinned to 50 km), four combinations of feature types, and 24 values of regularization multiplier, allowed us to develop an initial set of 288 models for each of *B*. *anthracis* and Bcbva from which to identify a best-fitting model for each pathogen species (Table 1). Overall, our three-step model selection framework (i.e., statistically significant pROC + low omission rate + low AICc) identified different features and regularization multipliers (i.e., parameters) for best models depending on the thinning scheme used. Models developed without thinning occurrences (*B*. *anthracis* n = 57, Bcbva n = 14) were overfitted, and had restricted predictive ability, especially for Bcbva (S1 Fig).

**Table 1:** Parametrization of selected *Bacillus anthracis* and Bcbva models. Parameters for models selected after a three-step selection framework (pROC, omission rates, and AICc), considering the different sets of occurrences and environments explored. Features: L = linear, LQ = linear + quadratic, LQP = linear + quadratic + product; RM: regularization multiplier; pROC: partial area under the Receiver Operating Characteristic; AICc: Akaike information criterion corrected for sample sizes.

Species	Occurrence thinning (km)	Occurrences	Selected features	Selected RM	Significance	Omission rate	AICc	Parameters
*B*. *anthracis*	0	57	LQP	10	<0.05	0.034	1267.62	9
*B*. *anthracis*	30	40	LQP	10	<0.05	0.1	943.57	8
*B*. *anthracis*	50	34	LQ	3	<0.05	0.059	792.12	13
Bcbva	0	14	L	0.8	<0.05	0.071	315.97	10
Bcbva	30	7	L	10	<0.05	0.14	166.05	2
Bcbva	50	5	L	0.6	<0.05	0.4	NA	9

**Fig 2:**
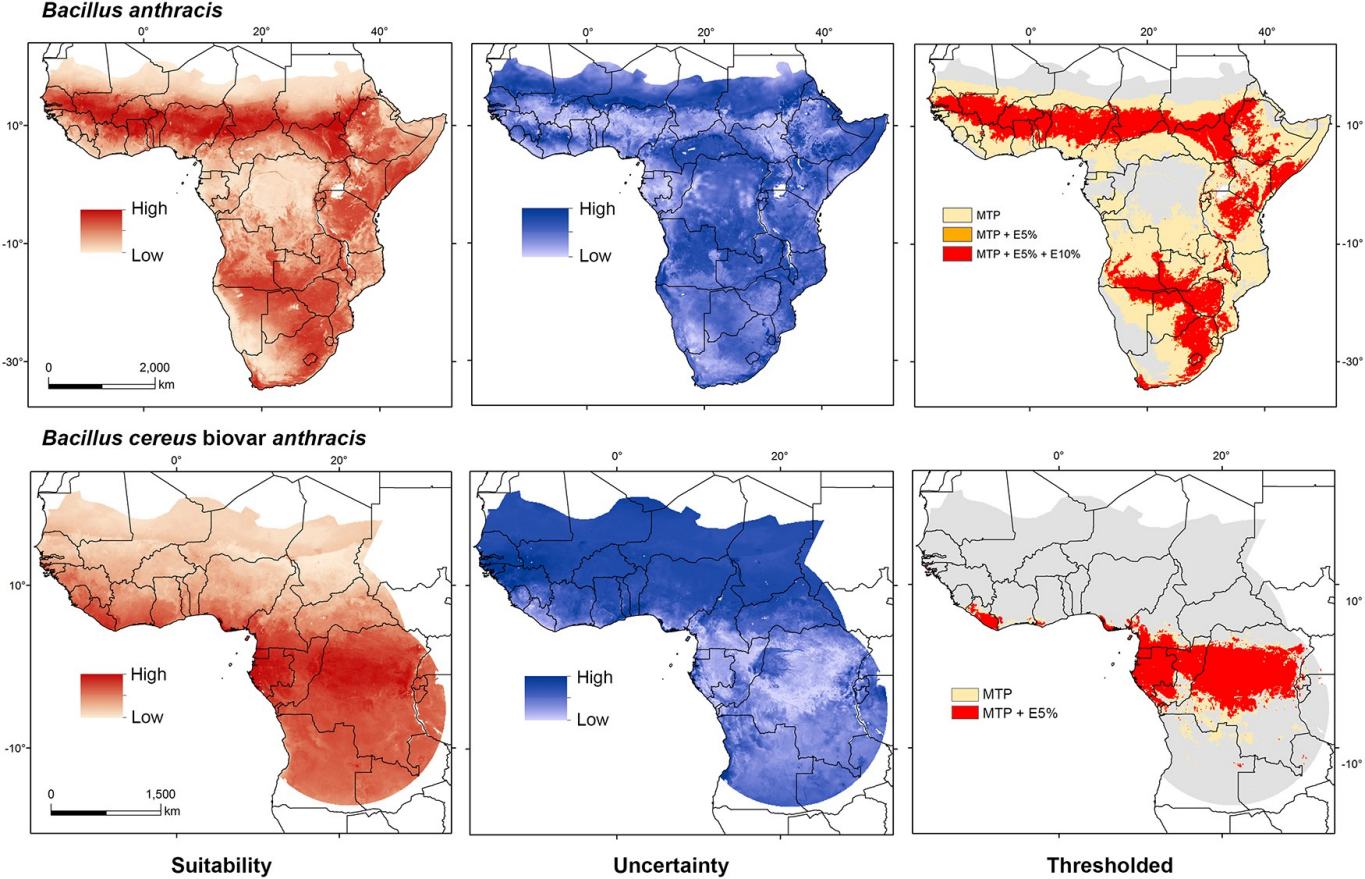
Model outputs for *Bacillus anthracis* and Bcbva. Models using 30 km thinned occurrence data are depicted for *B*. *anthracis* (top) and Bcbva (bottom), as maps of continuous suitability (left), uncertainty (center), and binary maps using different thresholds (right). For *B*. *anthracis* (top), binary maps were built using thresholds based on minimum training presence (MTP) in yellow, *E* = 5% in orange, and *E* = 10% in red. For Bcbva (bottom), binary maps were built using MTP (yellow) and *E* = 5% (red) thresholds. Maps were developed using shape files of Africa from the public domain repository of Natural Earth (http://www.naturalearthdata.com/) and built with ArcGIS 10.3 (ESRI Redlands, CA, USA).

Models for *B*. *anthracis* with 30 km spatial filtering yielded a broader prediction with less uncertainty (features = LQP, RM = 10; Table 1 and Fig 2, top). Binary models at MTP showed a larger area of suitability than binary models with other thresholds, despite minor differences between them (i.e., *E* = 5% and *E* = 10%, Fig 2, top right-hand panel). We found an east-west corridor of suitable areas for *B*. *anthracis* in the sub-Saharan region from Senegal, Sierra Leone, and southern Mali to Ethiopia. Binary maps with different threshold rules also showed consistent suitability in northern areas of Guinea, Côte d’Ivoire, Ghana, Nigeria, Cameroon, Central African Republic, South Sudan, and Uganda. Further, suitability was predicted for the majority of Togo and Benin, and southern parts of Burkina-Faso, Chad, and Sudan, all showing low levels of uncertainty (Fig 2, top). Other countries, such as Gabon, Equatorial Guinea, Eritrea, and Republic of the Congo, presented patchy areas of suitability for *B*. *anthracis* with varying levels of uncertainty (Fig 2, top). Consistent suitability from binary maps with low levels of uncertainty was also indicated for Zimbabwe; northern parts of Namibia, Botswana, South Africa, and Tanzania; and southern parts of Ethiopia, Somalia, Kenya, Zambia, and Angola (Fig 2, top).

**Fig 3:**
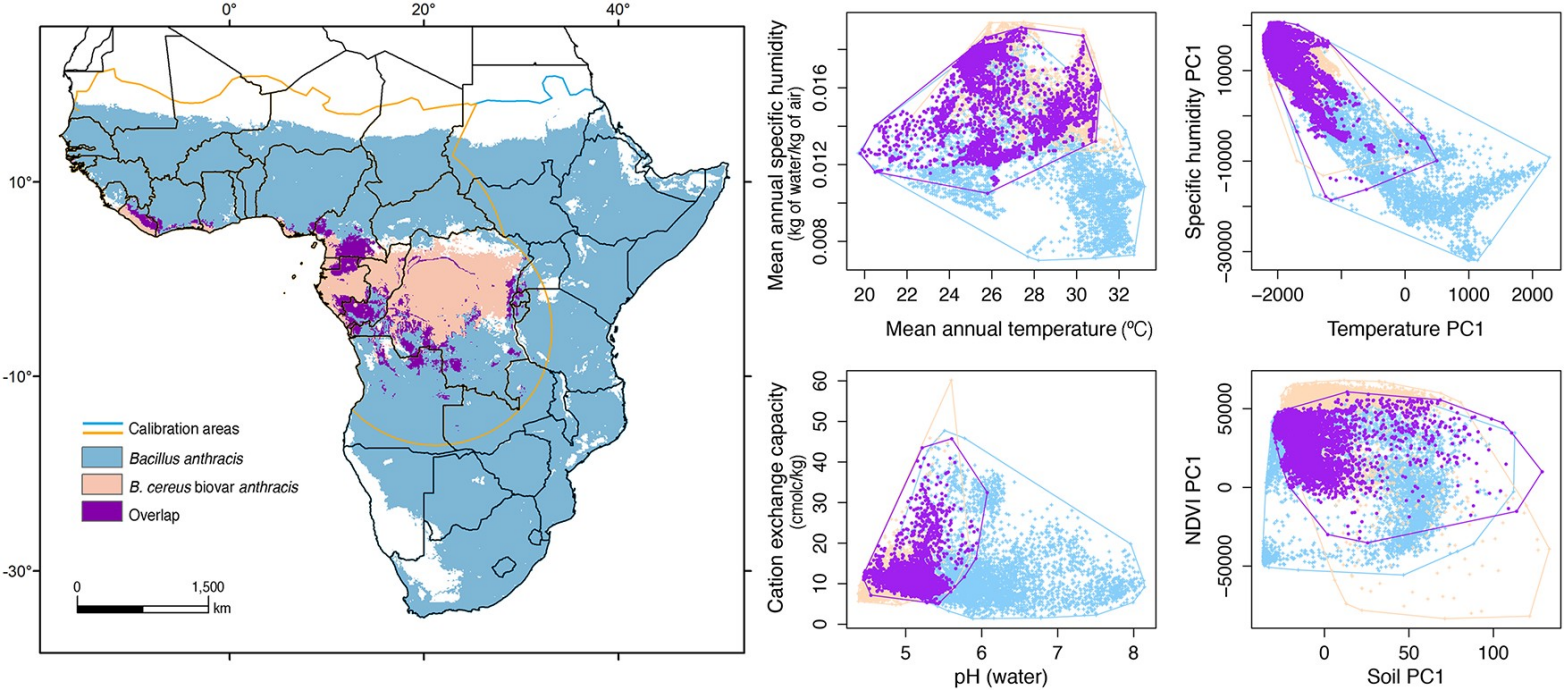
Overlapping regions for both pathogens. *Bacillus anthracis* (light blue) and Bcbva (light orange) suitability as relates to the Bcbva calibration area (orange line) using a minimum training presence threshold among the best performing models. Environments in these areas are characterized via a sample of 5000 points (right panels, same color scheme) considering four individual environmental variables (mean annual temperature, mean annual specific humidity, soil pH, and soil cation exchange capacity) and the first principal component (PC1) of the four environmental dimensions explored in this manuscript (temperature, specific humidity, vegetation greenness or NDVI, and soils). Maps were developed using shapefiles summarizing political borders of Africa from the public domain repository of Natural Earth (http://www.naturalearthdata.com/) and built with QGIS 2.18 ‘Las Palmas’.

For Bcbva we found, as in the previous case, that the model output with occurrences thinned at 30 km produced the best-performing model. Model uncertainty was low across the predicted region (Fig 2, bottom center). Binary maps were built using only MTP and *E* = 5% as thresholds considering the limited number of occurrences available; both thresholds depicted similar predictions with limited differences between them (Table 1 and Fig 2). Suitability for Bcbva with low uncertainty was indicated for Liberia, Gabon, Equatorial Guinea, Republic of the Congo, and southern regions of Ghana, Nigeria, and Cameroon by both binary rules (Fig 2, bottom); none of these areas was predicted as suitable for *B*. *anthracis*. Small areas of Guinea-Bissau showed patches of suitability, although with high uncertainty (Fig 2). Predictions for *B*. *anthracis* and Bcbva followed the overall suitability pattern described when using 50 km thinned occurrences (S2 Fig). Areas of overlapping suitability for *B*. *anthracis* and Bcbva within the Bcbva calibration area were identified using the MTP threshold in southern Cameroon and Republic of the Congo, northern Gabon and small areas of Burundi and Rwanda (Fig 3, left). These areas represent potential transitional environments from Bcbva to *B*. *anthracis* suitability and areas with the potential for pathogen co-occurrence. Contribution of each of the variables and response curves for the selected models can be found in the S1 File.

**Fig 4:**
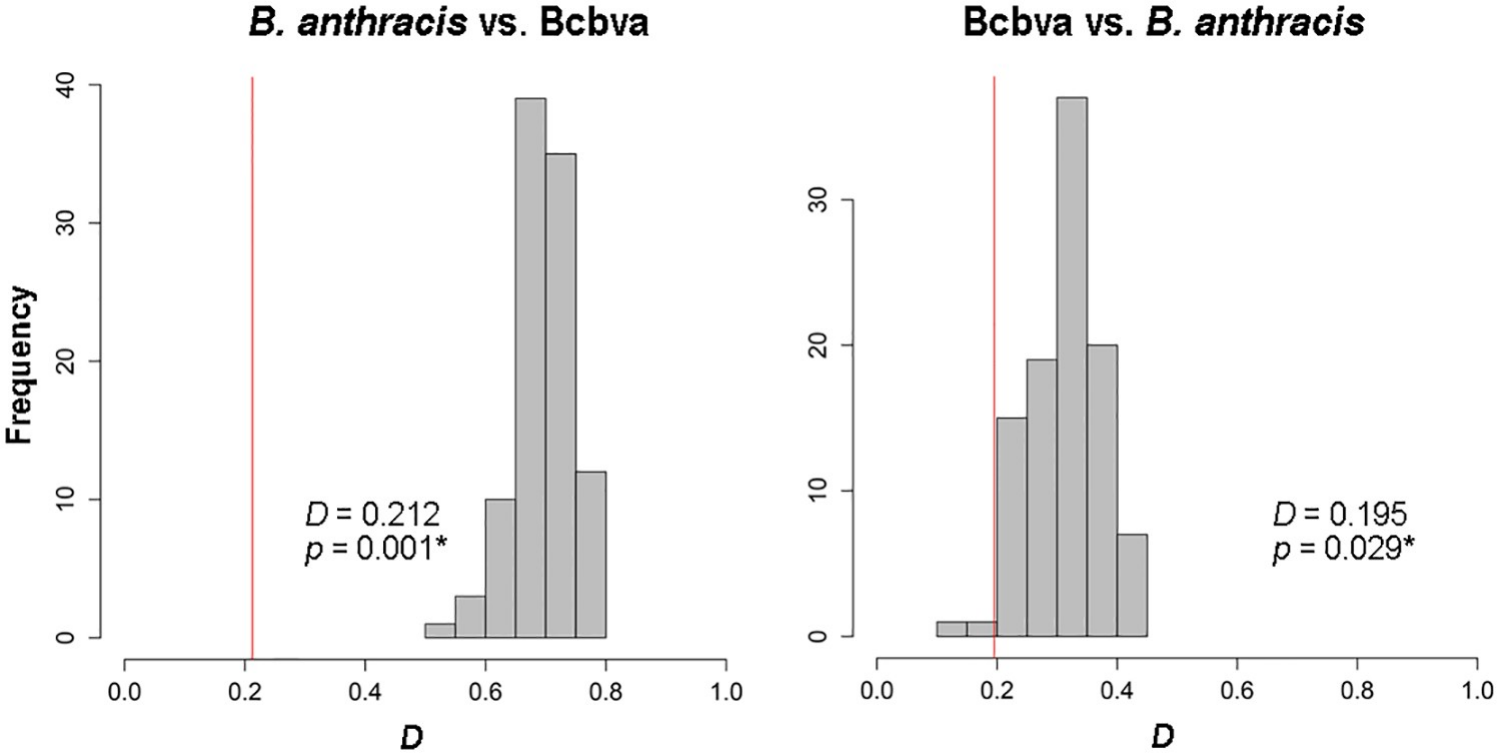
Background similarity test between *Bacillus anthracis* and Bcbva using all environments in geographic space. The comparison was done using all variables (i.e., 12 PCs) and calculating Schoener’s *D* statistic. Significant *p*-values (asterisk) indicate less similarity than expected when comparing to a random distribution.

**Fig 5:**
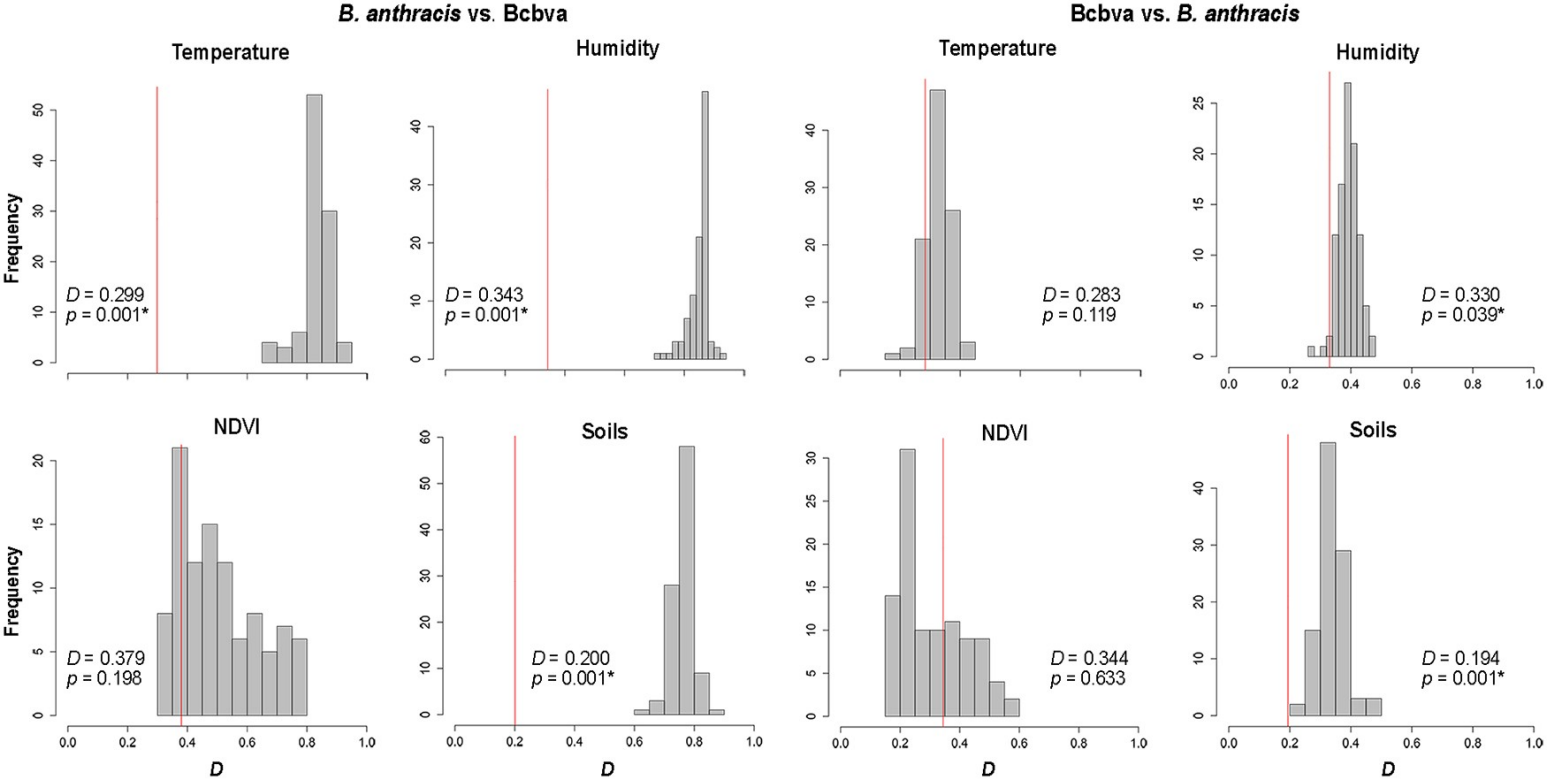
Background similarity tests between *Bacillus anthracis* and Bcbva for each individual environmental dimension. Comparisons were performed using three principal components for each environment, namely temperature, humidity, NDVI, and soils for *B*. *anthracis* vs. Bcbva (left) and Bcbva vs. *B*. *anthracis* (right). Significant *p*-values (asterisk) indicate less similarity than expected when comparing to a random distribution.

**Fig 6:**
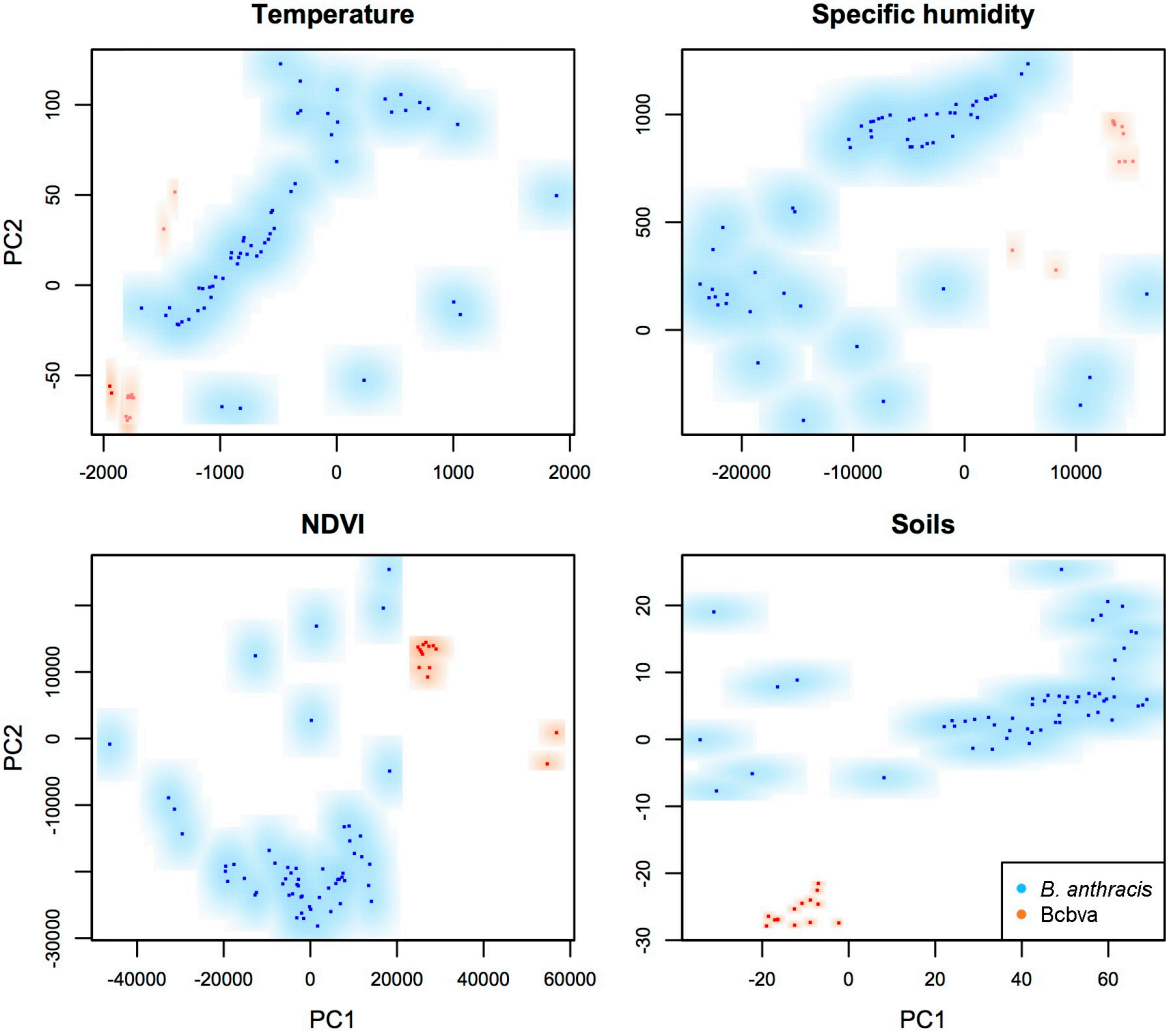
Kernel density plots around each point of *Bacillus anthracis* and Bcbva for temperature, humidity, NDVI, and soils depicted in the environmental space. Principal components one and two (PC1 and PC2) from each dimension were used to depict an environmental space to show regions occupied by *B*. *anthracis* (blue) and Bcbva (orange). Pathogens are using non-overlapping regions in each case.

Using the background similarity test with all variables (i.e., 12 PCs), we found statistical support for *B*. *anthracis* being less similar to Bcbva than expected at random (*D* = 0.212, *p* = 0.001), and for Bcbva being less similar to *B*. *anthracis* than expected at random (*D* = 0.195, *p* = 0.029) (Fig 4). In individual environmental dimensions, we found statistical support for *B*. *anthracis* as less similar to Bcbva than expected at random when comparing temperature, humidity, and soil conditions, but we could not reject the null hypothesis of similarity when comparing NDVI (Fig 5). The converse test (i.e., Bcbva vs. *B*. *anthracis*) failed to reject the hypothesis of similarity when comparing temperature and NDVI; however, we effectively rejected the hypothesis of similarity when comparing humidity (*D* = 0.330, *p* = 0.039) and soils (*D* = 0.194, *p* = 0.001) in which Bcbva was less similar to *B*. *anthracis* than expected at random (Fig 5). Comparisons between *B*. *anthracis* and Bcbva in environmental space failed to reject the hypothesis of similarity when comparing all variables and when comparing each individual environmental dimension (S3 and S4 Figs). Inspecting environments available for the two anthrax lineages inside each of the calibration areas, we can see that in no case do the environments represented in the two **M** regions overlap (i.e., for the two species; Fig 6 and S5 Fig).

## Discussion

This investigation is the first to map and assess the potential distribution of Bcbva, and to quantify similarities and differences between environments identified as suitable for *B*. *anthracis* and Bcbva across Africa. Model results provide valuable insight into the potential distribution of the emerging pathogen Bcbva, while identifying areas that may be at risk for coexistence of these two similar pathogens. We used an updated approach to model calibration and selection, generating multiple combinations of variables and parameter settings to identify the most robust model for prediction. Transparency in reporting model uncertainty and effects of different training data sets on model outputs provides a thorough set of information to ensure accurate model interpretation when observing local geographic areas [32,78]. Additionally, we focused our modeling effort on georeferenced confirmed cases of *B*. *anthracis* and Bcbva for model calibration. Confirmation is a critical component to robust modeling because several wildlife and livestock diseases can cause sudden death and present similar to anthrax, introducing the potential for spurious model results [79].

Predicted distributions of potentially suitable environments for *B*. *anthracis* in sub-Saharan Africa corresponded well to regions with confirmed and suspected anthrax cases. Specifically, model results identified suitable regions across the transition from humid tropical to semi-arid environments, from Senegal in West Africa across the continent to Ethiopia in East Africa (Figs 2 and 3). This transition zone consists of brush, grass, and savanna landscapes, highly suitable for livestock and wildlife grazing [80].

Model outputs identified suitable environments in locations with reports of suspected anthrax within Ethiopia, Kenya, Tanzania, and Uganda, despite having fewer georeferenced laboratory confirmed cases to use in model calibration (i.e., n = 40 for selected models; Fig 2). For example, model predictions indicated suitable environments for *B*. *anthracis* in and around Serengeti National Park in Tanzania, where Lembo et al (2011), found several animals testing seropositive for anthrax [81]. Model outputs for Kenya included suitability corresponding to several locations where suspected anthrax outbreaks occurred, such as southern Samburu District, where livestock, Grevy’s zebras, and plains zebras died from suspected anthrax in 2005–2006 [82]. Similarly, Uganda experienced devastating outbreaks of anthrax among wildlife in 2004–2005 and 2010 [83,84]. The burden of anthrax on both countries prompted a collaborative assembly of medical, veterinary, and wildlife experts to identify anthrax as a top priority, when implementing a One Health Zoonotic Disease Prioritization Tool in 2015 for Kenya [85] and in 2017 for Uganda [86].

Models identified potentially suitable environments for *B*. *anthracis* across areas in southern Africa, including parts of Zambia, Malawi, Mozambique, Zimbabwe, Swaziland, Lesotho, South Africa, Botswana, Namibia, and Angola (Fig 2). Most notable are areas within several wildlife reserves, where enzootic and epizootic anthrax are known to impact wildlife. Best-known are Kruger National Park in South Africa and Etosha National Park in Namibia [87], where seminal studies on relationships between anthrax and soil pH and calcium were conducted [88]. Model outputs suggested suitable environments across much of both parks, in addition to the Caprivi region in the panhandle of Namibia, where a large anthrax outbreak in hippopotami occurred in 2017 [89]. Additional georeferenced laboratory-confirmed locations will help to refine model results, providing valuable information for model calibration and facilitating a more complete representation of suitable environments for anthrax across sub-Saharan Africa, particularly in transition zones from savanna environments into humid forested areas. While aggregate empirical evidence suggests that *B*. *anthracis* is not present within humid forest environments, additional sampling in transition zones may reveal a slightly broader environmental breadth than is appreciated currently, particularly given the present focus on livestock and savanna wildlife deaths.

On the other hand, model outputs for Bcbva suggested a more compact environmental distribution than *B*. *anthracis*, limited to areas with humid tropical forested environments (Fig 2). The predicted distribution of Bcbva indicated potential suitability across much of the Congo Basin and in patches along the coast of the Gulf of Guinea in West Africa. Limited calibration data could be constraining model outputs to environments specific to sampling schemes, but this investigation provides a first look at potential distributions of this emerging pathogen. An incomplete picture of uncertainty in model predictions for Bcbva is likely owing to the smaller number of calibration points (i.e., n = 7 in selected models, Fig 2), which is also the reason why we avoided extrapolation of final Bcbva models to a broader region than the calibration zone, depicting potential overlap between both pathogens only inside the Bcbva calibration area (Fig 3). Considering the disjunct geographic distribution of Bcbva isolates, humid tropical forested areas identified as suitable provide a reference by which further surveillance and investigation can be guided. Finally, although the possibility of previous unknown Bcbva outbreaks in other regions is present, the long-time surveillance efforts for *B*. *anthracis* in Africa would have found Bcbva outside forested environments at earlier times, a situation never reported before the 2000’s [20].

Background similarity results indicated significant differences between the ecological niches of *B*. *anthracis* and Bcbva (Fig 4), although only partially when comparing individual variables between Bcbva and *B*. *anthracis* (Fig 5). While this result may seem counter-intuitive, the outcome is clear, when considered in the context of comparing two species where one niche is relatively known (i.e., *B*. *anthracis*), while the other is only incompletely characterized (i.e., Bcbva); also, the imbalance in sample sizes may create differences in statistical power and consequent ability to detect real differences. In this context, our results suggest with confidence that suitable temperature, humidity, and soils environments for the well-characterized *B*. *anthracis* are less similar than expected to those observed for Bcbva, indicating that the humid forested environments from which Bcbva has been isolated are not associated with historical or current observations of *B*. *anthracis* in sub-Saharan Africa. Additional georeferenced locations of laboratory-confirmed Bcbva cases or isolates, particularly in transition zones between humid forest and savanna environments, will contribute to a more robust characterization of the niche of Bcbva, improving model predictions and comparisons between environments suitable for Bcbva vs. *B*. *anthracis*.

Soils and humidity values were a significant differentiating variable when comparing *B*. *anthracis* to Bcbva and when comparing Bcbva to *B*. *anthracis*. Associations between elevated soil pH values and *B*. *anthracis* are a key factor associated with spore survival and identification of geographic areas at risk for anthrax from *B*. *anthracis* [7,16], and our analysis suggests that soil attributes may continue to provide important information, when assessing risk for *B*. *anthracis* vs. Bcbva. Furthermore, humidity has been also incriminated as another of the environmental determinants of sporulation for *B*. *anthracis* and its role in the maintenance of spores for Bcbva should also be explored empirically [7,26].

On the other hand, NDVI proved not to be a differentiating variable when comparing *B*. *anthracis* to Bcbva and when comparing Bcbva to *B*. *anthracis*. This result was interesting, given the differences between spectral signatures from dry savanna environments vs. humid forested environments [56]. One possibility is that distribution of variance of NDVI values in this data set occurred across a greater number of PCs than we included in this analysis. The first three PCs explained 91.5% of the variance for NDVI values, compared to >97% explained variance for temperature, humidity, and soils, respectively. A refined analysis of NDVI and perhaps exploration of enhanced vegetation indices, in the context of greater numbers of georeferenced occurrence data for both pathogens, could reveal differences not observed here. While not explored in this study, additional abiotic factors such as wind strength and direction might also impact the distributions of *B*. *anthracis* and Bcbva. Although wind clearly drives distributions of spore-forming bacteria, further assessment of its role as a macro-ecological determinant of anthrax outbreaks remains to be conducted [7,15,16].

Additionally, results from background similarity tests using the kernel density approach across a gradient of environments did not yield results consistent with those of the geographic-space and binary model comparisons; this was explained when analyzing the environments of the occurrences for *B*. *anthracis* and Bcbva as shown in Fig 5 and S5 Fig. Without an overlap, the statistical power of environmental-space comparisons to detect differences is nil: one never can “see” one species using or not using the environments used by the other species.

In sum, the full niche breadth of Bcbva remains unknown, particularly whether Bcbva occurs beyond humid forested environments within ecological transition zones moving toward drier savanna environments common to *B*. *anthracis*. A logical next step is to investigate for presence of Bcbva in transition zones from forested to savanna environments in West Africa where our models based on MTP thresholds showed a potential overlap for both pathogens (Fig 3). The information obtained from the analyses presented here can be used to identify areas of interest for increased surveillance of animal deaths compatible with Bcbva to spur additional sampling and testing, while also considering the similarities regarding virulence between *B*. *anthracis* and Bcbva [26]. In this context, recent research efforts have started to identify Bcbva-specific proteins with immunogenic potential expressed by Bcbva and not by *B*. *anthracis* (i.e., pXO2-60, [90]) which can be used as a first antibody-based discriminatory diagnostic tool for in-field serological surveys; notably, transition zones where both pathogens may be present could occur in multiple regions in sub-Saharan Africa, including in Cameroon, and the southwestern of the Republic of Congo (Fig 3). As additional information becomes available for Bcbva, it will be important to investigate this hypothesis further to determine whether humans and animals in these transition zones are at greater risk for exposure to this emerging pathogen.

## Supporting information

S1 Table Occurrences of *Bacillus anthracis* and Bcbva used in the study. (XLSX)

S2 Table Environmental dimensions considered in the present study. (DOCX)

S1 File Details on methods, evaluation metrics, R packages used in the manuscript, variable contribution, and response curves. (DOCX)

S1 Fig Model outputs for *Bacillus anthracis* and Bcbva (no thinning). Models using all occurrences (no thinning) are depicted for *B*. *anthracis* (top) and Bcbva (bottom), as maps of continuous suitability (left), uncertainty (center), and binary maps using different thresholds (right). Maps were developed using shape files of Africa from the public domain repository of Natural Earth (http://www.naturalearthdata.com/) and build with ArcGIS 10.3 (ESRI Redlands, CA, USA).
(TIF)

S2 Fig Model outputs for *Bacillus anthracis* and Bcbva (50 km thinning). Models using 50 km thinned records are depicted for *B*. *anthracis* (top) and Bcbva (bottom), as maps of continuous suitability (left), uncertainty (center), and binary maps using different thresholds (right). Maps were developed using shape files of Africa from the public domain repository of Natural Earth (http://www.naturalearthdata.com/) and build with ArcGIS 10.3 (ESRI Redlands, CA, USA).
(TIF)

S3 Fig Background similarity test in environmental space between *Bacillus anthracis* and Bcbva. Comparison was performed calculating Schoener’s *D* statistic on kernel density functions on an environmental space delimited by the first two principal components of the overall set. Results are depicted for *B*. *anthracis* vs. Bcbva (left) and Bcbva vs. *B*. *anthracis* (right).
(TIF)

S4 Fig Background similarity tests in environmental space for individual variables between *Bacillus anthracis* and Bcbva. Comparison was performed calculating Schoener’s *D* statistic on kernel density functions on an environmental space delimited by the first two principal components of each corresponding set (i.e., temperature, humidity, NDVI, and soils). Results are depicted for *B*. *anthracis* vs. Bcbva (left) and Bcbva vs. *B*. *anthracis* (right).
(TIF)

S5 Fig Kernel density plots around each point of *Bacillus anthracis* and Bcbva in the environmental space. Principal components one and two (PC1 and PC2) from all the available environments (i.e., 12 variables) were used to depict an environmental space to show regions occupied by *B*. *anthracis* (blue) and Bcbva (red). Pathogens are using non-overlapping regions.
(TIF)
