# Supplementary material for: Potential distributions of Bacillus anthracis and Bacillus cereus biovar anthracis causing anthrax in Africa
Source: PLoS Negl Trop Dis. 2020 Mar 9;14(3):e0008131. doi: 10.1371/journal.pntd.0008131 (PMC7082064; doi:10.1371/journal.pntd.0008131)
Supplement: S2 Table — (DOCX) [file pntd.0008131.s002.docx]

**Potential distributions of *Bacillus anthracis* and *Bacillus cereus* biovar *anthracis* causing anthrax in Africa**

Daniel Romero-Alvarez, A. Townsend Peterson, Johanna S. Salzer, Claudia Pittiglio, Sean Shadomy, Rita Traxler, Antonio R. Vieira, William A. Bower, Henry Walke, Lindsay P. Campbell

**S2 Table. Environmental dimensions considered in the present study.**

We explored the suitability of *B. anthracis* and Bcbva in four environmental dimensions: temperature, humidity (climate), vegetation indices, and soils.

| **Enviromental dimension (Variables)** | **Spatial and**  **Temporal resolution** | **Source / Reference** | **Availability** |
| --- | --- | --- | --- |
| **Temperature (9 layers)*** | **5 arc minutes / 2000-2010** | MERRAclim  [1] | [https://datadryad.org//resource/doi:10.5061/dryad.s2v81](https://datadryad.org/resource/doi:10.5061/dryad.s2v81) |
| BIO1 | Annual mean temperature |  |  |
| BIO2 | Mean diurnal range temperature |  |  |
| BIO3 | Isothermality |  |  |
| BIO4 | Temperature seasonality |  |  |
| BIO5 | Maximum temperature of the warmest month |  |  |
| BIO6 | Minimum temperature of the coldest month |  |  |
| BIO7 | Temperature annual range |  |  |
| BIO10 | Mean temperature of warmest quarter |  |  |
| BIO11 | Mean temperature of coldest quarter |  |  |
| **Humidity  (6 layers)*** | **5 arc minutes / 2000-2010** | MERRAclim  [1] | [https://datadryad.org//resource/doi:10.5061/dryad.s2v81](https://datadryad.org/resource/doi:10.5061/dryad.s2v81) |
| BIO12 | Annual mean specific humidity |  |  |
| BIO13 | Specific humidity of most humid month |  |  |
| BIO14 | Specific humidity of least humid month |  |  |
| BIO15 | Specific humidity seasonality |  |  |
| BIO16 | Specific humidity mean of most humid quarter |  |  |
| BIO17 | Specific humidity mean of least humid quarter |  |  |
| **Vegetation index  (299 layers)** | **500 m / 2005-2017** |  |  |
| NDVI** | MOD13A1 product version 6 | Moderate Resolution Imaging Spectroradiometer (MODIS) [2] | <https://lpdaac.usgs.gov/data_access/data_pool> |
| **Soils  (6 layers)** | **250 meters / 2012-2016** |  |  |
| CECSOL† | Cation exchange capacity of soils at two depths | SoilGrids  [3] | <https://soilgrids.org/#!/?layer=TAXNWRB_250m&vector=1> |
| ORCDRA† | Soil organic carbon content at two depths |  |  |
| PHIHOX† | Soil pH x 10 in H2O at two depths |  |  |

*Bioclimatic variables combining information from temperature and humidity (BIO-8, BIO-9, BIO-18 and BIO-19) were not included in our analysis.

**Normalized Difference Vegetation Index

†Depths considered for each variable: 0-5 cm and 5-15 cm

**References**

1. Vega GC, Pertierra LR, Olalla-Táraga MÁ. MERRAclim, a high-resolution global dataset of remotely sensed bioclimatic variables for ecological modelling. Sci Data. 2017;4: 170078.

2. Horning N, Robinson JA, Sterling EJ, Turner W, Spector S. Remote Sensing for Ecology and Conservation: A Handbook of Techniques. New York: Oxford University Press; 2010.

3. Hengl T, Mendes de Jesus J, Heuvelink GBM, Ruiperez Gonzalez M, Kilibarda M, Blagotić A, et al. SoilGrids250m: global gridded soil information based on machine learning PLoS ONE. 2017;12: e0169748.
