# Supplementary material for: Potential distributions of Bacillus anthracis and Bacillus cereus biovar anthracis causing anthrax in Africa
Source: PLoS Negl Trop Dis. 2020 Mar 9;14(3):e0008131. doi: 10.1371/journal.pntd.0008131 (PMC7082064; doi:10.1371/journal.pntd.0008131)
Supplement: S1 File — (DOCX) [file pntd.0008131.s003.docx]

**Potential distributions of *Bacillus anthracis* and *Bacillus cereus* biovar *anthracis* causing anthrax in Africa**

Daniel Romero-Alvarez, A. Townsend Peterson, Johanna S. Salzer, Claudia Pittiglio, Sean Shadomy, Rita Traxler, Antonio R. Vieira, William A. Bower, Henry Walke, Lindsay P. Campbell

**S1 File. Details on methods and evaluation metrics, R packages used in the manuscript, variable contribution, and response curves.**

- **Details on methods**
  - Ecological niche models
  - Evaluation framework
- **S1 File Table 1. Packages of the programming language R [26] implemented in different sections of the manuscript.**
- **S1 File Table 2. Summary of variable contribution for the selected model of *Bacillus anthracis* (i.e. Thinned to 30 km).**
- **S1 File Table 3. Summary of variable contribution for the selected model of *Bacillus cereus* biovar *anthracis* (i.e. Thinned to 30 km).**
- **S1 File Fig 1. Response curves considering the individual influence of different variables to the selected model (i.e., Thinned to 30 km) for *Bacillus anthracis*.**
- **S1 File Fig 2. Response curves considering the individual influence of different variables to the selected model (i.e., Thinned to 30 km) for *Bacillus cereus* biovar *anthracis*.**
- **Supplementary material references**

**Detail on methods**

**Ecological niche models**

Currently, there is a plethora of algorithms to develop ecological niche models [1–3], and some methodologies suggest the use of multiple algorithms applied to the same data to generate model ensembles summarizing modeling results [4–6]. However, recent publications demonstrated the absence of an all-purpose algorithm to be applied in ecological niche modeling experiments [2,3] and additional investigations revealed a lack of improvement in model results using model ensemble techniques [6,7]. For modeling the suitability of *B. anthracis* and Bcbva in Africa we selected Maxent (version 3.3.3k), a machine-learning algorithm based on presence data and background information obtained from a defined calibration region [8,9]. Maxent allows an extensive array of tuning options to generate multiple model outputs exploring different sets of parametrizations [7,10,11]. We explored different combinations of Maxent features (i.e., linear [L], linear+quadratic [LQ], linear+quadratic+product [LQP], and linear+quadratic+product+threshold+hinge [LQPTH]) and regularization coefficients (i.e., 0.1 to 2, by increments of 0.1, and also coefficient values of 3, 5, 7 and 10) which agrees with the need for research-oriented individualized criteria for the development of accurate models [10,12,13]. An in depth discussion of Maxent and its parameterization capabilities can be found in [14,15].

**Evaluation framework**

To evaluate *B. anthracis* generated models, we randomly split all available occurrences and keep 50% for model calibration, and used the other 50% for model evaluation. We used a three-step process to select our final models, pondering statistical significance, performance, and complexity [16,17]. We assessed statistical significance among predicted models using the partial area under the Receiver Operating Characteristic (pROC) as described in [17,18]. This metric is a modification of the customary ROC area under the curve (AUC) and relies on bootstrapping (here, 1,000 iterations), and the ratio between correct predictions (i.e., occurrences accurately predicted by the model) and the proportion of the area with the lowest omission errors. The ratio ranges from <= 1 (*p* > 0.05) to > 1 (*p* < 0.05); that is, predictions indistinguishable and those better than random respectively [17,18]. Significant models under the pROC statistic were explored under the scope of omission rates considering a threshold rule of 5% [19]. Omission rates determine the ability of the predicted area of the model to successfully identify evaluation occurrences (true positives) by accounting for those occurrences of the evaluation dataset that were omitted (false negatives) [19,20]; thus, low values of omission rate allow us to select the best performing models. Statistical significant models with lower omission rates were discriminated using the Akaike information criteria corrected for sample sizes (AICc), a metric accounting for model complexity with lower values representing models with low complexity and good fit to the data [20–22]. Model selection using this framework has been implemented within the kuenm package for R, available at <https://github.com/marlonecobos/kuenm> [16].

For developing ecological niche models in settings of low sample sizes (e.g., less than 25 points), as is the case of Bcbva, models should be built using a leave-one-out approach [23–25] Here, for each modeling round, we set aside one occurrence and test the ability of the model to predict this occurrence using all the other calibration points, thus, being *n* the total number of occurrences, we developed *n*-1 models until all occurrences have been evaluated. We calculated *p* values for each model manually using the software provided in [23] (i.e., PvalueCompute.exe). Then, we used the ENMeval package in R [20] to calculate omission rates using a minimum training presence (MTP) threshold rule [19,23]. As in the case of *B. anthracis*, we used these omission rates as a measure of performance and AICc as a measure of complexity; we were unable to calculate AICc values for Bcbva models thinned to 50 km because of the limited number of occurrences (n = 5) and the high number of parameters (i.e., nine) in the best performing model (i.e., lowest omission rate; see Table 1 in main text) [21,22].

**S1 File Table 1. Packages of the programming language R [26] implemented in different sections of the manuscript.**

| **Package** | **Application** | **Reference** | **Availability** |
| --- | --- | --- | --- |
| ENMeval | Model calibration and evaluation metrics. Omission rates for Bcbva models. | [20] | <https://github.com/bobmuscarella/ENMeval> |
| RStoolbox | Principal component analysis. | [27] | <https://cran.r-project.org/web/packages/RStoolbox/RStoolbox.pdf> |
| kuenm | Model calibration, selection and calculation of evaluation metrics for *B. anthracis*. | [16] | <https://github.com/manubio13/ku.enm> |
| ENMtools (R implementation) | Niche similarity analysis. Background similarity test and kernel density functions (i.e., ecospat). | [28] | <https://github.com/danlwarren/ENMTools>. |
| raster | Manipulation of raster files: crop, resample, mask, etc. | [29] | <https://cran.r-project.org/web/packages/raster/vignettes/Raster.pdf> |
| rts | Download of MODIS raster files (see Supp. table 2). | [30] | <https://cran.r-project.org/web/packages/rts/rts.pdf> |
| Plot_ConvexHull | Function that develops convex hull in plots (e.g., Fig 5). | [31] | <https://chitchatr.wordpress.com/2011/12/30/convex-hull-around-scatter-plot-in-r/> |

**S1 File Table 2. Summary of variable contribution for the selected model of *Bacillus anthracis* (i.e. Thinned to 30 km).** Variables are ordered from high to low contributions. The principal component one (PC1) from the NDVI variable set is contributing the most for the model.

| **Variable** | **Percent contribution (%)** |
| --- | --- |
| NDVI PC1 | 62.5 |
| NDVI PC2 | 7 |
| Humidity PC2 | 7 |
| Soil PC2 | 5.6 |
| Soil PC1 | 5.3 |
| Temperature PC3 | 5.2 |
| Humidity PC1 | 3.7 |
| Humidity PC3 | 1.4 |
| NDVI PC3 | 0.9 |
| Temperature PC1 | 0.7 |
| Soil PC3 | 0.6 |
| Temperature PC2 | 0 |

**S1 File Table 3. Summary of variable contribution for the selected model of *Bacillus cereus* biovar *anthracis* (i.e. Thinned to 30 km).** Variables are ordered from high to low contributions. The principal component two (PC2) from the soil variable set is contributing the most for the model.

| **Variable** | **Percent contribution (%)** |
| --- | --- |
| Soil PC2 | 72.4 |
| Temperature PC1 | 20.7 |
| Humidity PC1 | 4.5 |
| Soil PC1 | 2.4 |
| Temperature PC2 | 0 |
| Temperature PC3 | 0 |
| NDVI PC3 | 0 |
| NDVI PC2 | 0 |
| NDVI PC1 | 0 |
| Humidity PC3 | 0 |
| Humidity PC2 | 0 |
| Soil PC3 | 0 |

**S1 File Fig 1. Response curves considering the individual influence of different variables to the selected model (i.e., Thinned to 30 km) for *Bacillus anthracis*.**

**S1 File Fig 2. Response curves considering the individual influence of different variables to the selected model (i.e., Thinned to 30 km) for *Bacillus cereus* biovar *anthracis*.**

**Supplementary material references**

1. Escobar LE, Qiao H, Cabello J, Peterson AT. Ecological niche modeling re-examined: a case study with the Darwin’s fox. Ecol Evol. 2018;8: 4757–4770.

2. Qiao H, Feng X, Escobar LE, Peterson AT, Soberón J, Zhu G, et al. An evaluation of transferability of ecological niche models. Ecography. 2018;in press.

3. Qiao H, Soberon J, Peterson AT. No silver bullets in correlative ecological niche modelling: Insights from testing among many potential algorithms for niche estimation. Methods Ecol Evol. 2015;6: 1126–1136.

4. Marmion M, Parviainen M, Luoto M, Heikkinen RK, Thuiller W. Evaluation of consensus methods in predictive species distribution modelling. Divers Distrib. 2009;15: 59–69.

5. Araújo MB, Whittaker RJ, Ladle RJ, Erhard M. Reducing uncertainty in projections of extinction risk from climate change. Glob Ecol Biogeogr. 2005;14: 529–538.

6. Zhu GP, Peterson AT. Do consensus models outperform individual models? Transferability evaluations of diverse modeling approaches for an invasive moth. Biol Invasions. 2017;19: 2519–2532.

7. Zhu G, Fan J, Peterson AT. *Schistosoma japonicum* transmission risk maps at present and under climate change in mainland China. PLoS Negl Trop Dis. 2017;11: e0006021.

8. Phillips SJ, Anderson RP, Schapire RE. Maximum entropy modeling of species geographic distributions. Ecol Modell. 2006;190: 231–259.

9. Phillips SJ, Dudík M. Modeling of species distributions with Maxent: new extensions and a comprehensive evaluation. Ecography. 2008;31: 161–175.

10. Radosavljevic A, Anderson RP. Making better Maxent models of species distributions: complexity, overfitting and evaluation. J Biogeogr. 2014;41: 629–643.

11. Alkishe AA, Peterson AT, Samy AM. Climate change influences on the potential geographic distribution of the disease vector tick *Ixodes ricinus*. PLoS ONE. 2017;12: e0189092.

12. Romero-Alvarez D, Escobar LE, Varela S, Larkin DJ, Phelps NBD. Forecasting distributions of an aquatic invasive species (*Nitellopsis obtusa*) under future climate scenarios. PLoS ONE. 2017;12: e0180930.

13. Peterson AT. Mapping Disease Transmission Risk: Enriching Models Using Biogeography and Ecology. Enriching Models Using Biogeography and Ecology. Baltimore: Johns Hopkins University Press; 2014.

14. Elith J, Phillips SJ, Hastie T, Dudík M, Chee YE, Yates CJ. A statistical explanation of Maxent for ecologists. Divers Distrib. 2011;17: 43–57.

15. Merow C, Smith MJ, Silander JA. A practical guide to Maxent for modeling species’ distributions: what it does, and why inputs and settings matter. Ecography. 2013;36: 1058–1069.

16. Cobos ME, Peterson AT, Barve N, Osorio-Olvera L. kuenm: an R package for detailed development of ecological niche models using Maxent. PeerJ. 2019;7: e6281.

17. Peterson AT, Papeş M, Soberón J. Rethinking receiver operating characteristic analysis applications in ecological niche modeling. Ecol Modell. 2008;213: 63–72.

18. Peterson AT. Niche modeling: model evaluation. Biodivers Informatics. 2012;8: 41.

19. Peterson AT, Soberón J, Pearson RG, Anderson RP, Martínez-Meyer E, Nakamura M, et al. Ecological Niches and Geographic Distributions. New Jersey: Princeton University Press; 2011.

20. Muscarella R, Galante PJ, Soley-Guardia M, Boria RA, Kass JM, Uriarte M, et al. ENMeval: an R package for conducting spatially independent evaluations and estimating optimal model complexity for Maxent ecological niche models. Methods Ecol Evol. 2014;5: 1198–1205.

21. Burnham KP, Anderson DR, Huyvaert KP. AIC model selection and multimodel inference in behavioral ecology: some background, observations, and comparisons. Behav Ecol Sociobiol. 2011;65: 23–35.

22. Warren DL, Seifert SN. Ecological niche modeling in Maxent: the importance of model complexity and the performance of model selection criteria. Ecol Appl. 2011;21: 335–342.

23. Pearson RG, Raxworthy CJ, Nakamura M, Peterson AT. Predicting species distributions from small numbers of occurrence records: a test case using cryptic geckos in Madagascar. J Biogeogr. 2007;34: 102–117.

24. Galante PJ, Alade B, Muscarella R, Jansa SA, Goodman SM, Anderson RP. The challenge of modeling niches and distributions for data-poor species: a comprehensive approach to model complexity. Ecography. 2018;41: 726–736.

25. Shcheglovitova M, Anderson RP. Estimating optimal complexity for ecological niche models: a jackknife approach for species with small sample sizes. Ecol Modell. 2013;269: 9–17.

26. R Core Team. R: A language and environment for statistical computing. Vienna, Austria: R Foundation for Statistical Computing; 2018. Available at: <https://www.R-project.org/>

27. Leutner B, Horning N, Schwalb-Willmann J, Hijmans RJ. Package ‘RStoolbox.’ 2019. Available at: <https://cran.r-project.org/web/packages/RStoolbox/RStoolbox.pdf>

28. Warren DL, Glor RE, Turelli M. ENMTools: a toolbox for comparative studies of environmental niche models. Ecography. 2010;33: 607–611.

29. Hijmans RJ, van Etten J, Cheng J, Mattiuzzi M, Sumner M, Greenberg JA, et al. Package ‘raster’: geographic data analysis and modeling. 2018. Available: <https://cran.r-project.org/web/packages/raster/raster.pdf>

30. Naimi B. Package 'rts': Raster Time Series Analysis. 2018. Available: <https://cran.r-project.org/web/packages/rts/rts.pdf>

31. Ken Takagi. Convex Hull around scatter plot in R | Chit Chat R. Available: <https://chitchatr.wordpress.com/2011/12/30/convex-hull-around-scatter-plot-in-r/>
