# Supplementary figures and images for: Potential distributions of Bacillus anthracis and Bacillus cereus biovar anthracis causing anthrax in Africa
Source: PLoS Negl Trop Dis. 2020 Mar 9;14(3):e0008131. doi: 10.1371/journal.pntd.0008131 (PMC7082064; doi:10.1371/journal.pntd.0008131)

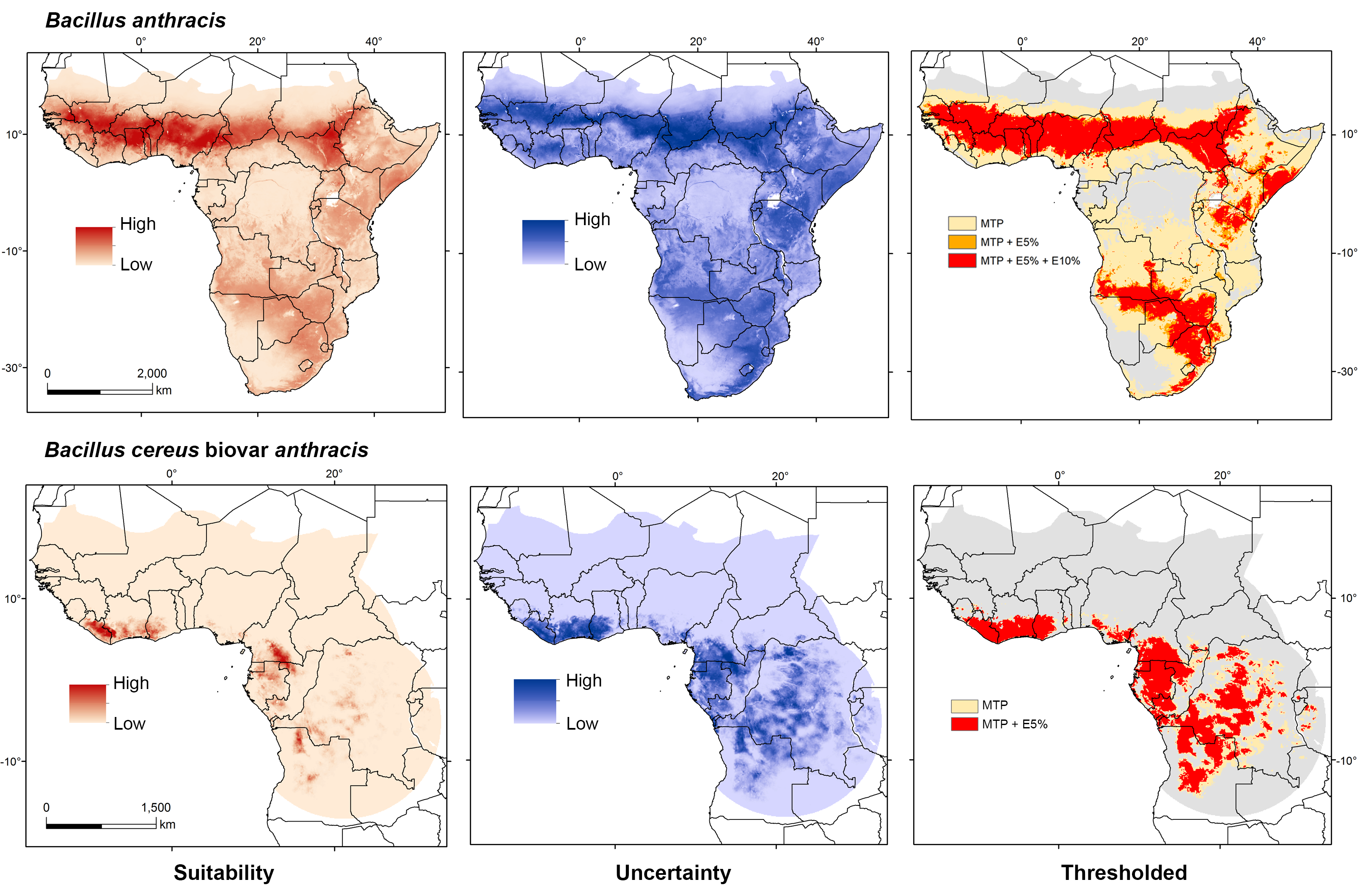

Supplement: S1 Fig — Models using all occurrences (no thinning) are depicted for B. anthracis (top) and Bcbva (bottom), as maps of continuous suitability (left), uncertainty (center), and binary maps using different thresholds (right). Maps were developed using shape files of Africa from the public domain repository of Natural Earth (http://www.naturalearthdata.com/) and build with ArcGIS 10.3 (ESRI Redlands, CA, USA). (TIF) [file pntd.0008131.s004.tif]

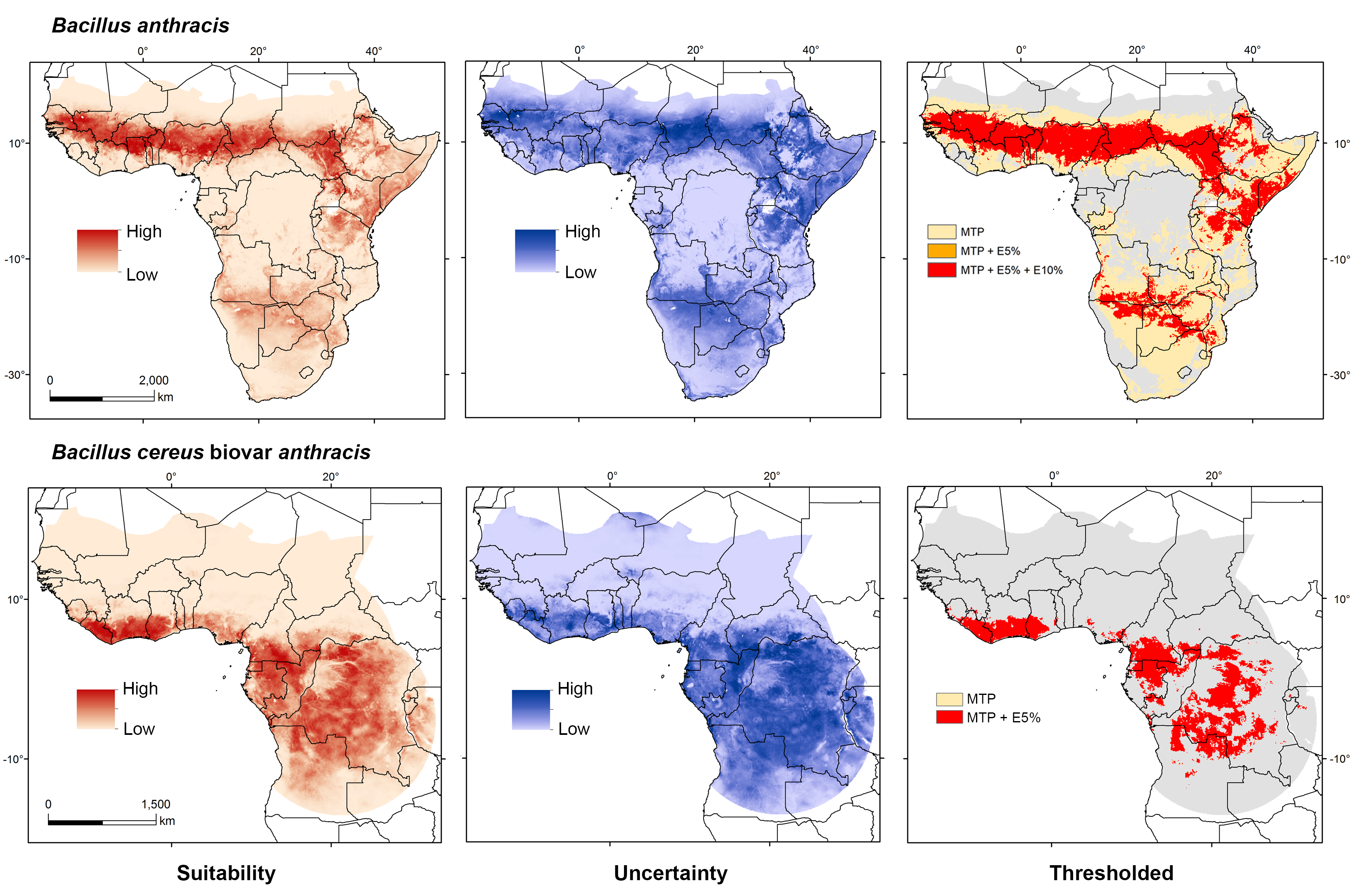

Supplement: S2 Fig — Models using 50 km thinned records are depicted for B. anthracis (top) and Bcbva (bottom), as maps of continuous suitability (left), uncertainty (center), and binary maps using different thresholds (right). Maps were developed using shape files of Africa from the public domain repository of Natural Earth (http://www.naturalearthdata.com/) and build with ArcGIS 10.3 (ESRI Redlands, CA, USA). (TIF) [file pntd.0008131.s005.tif]

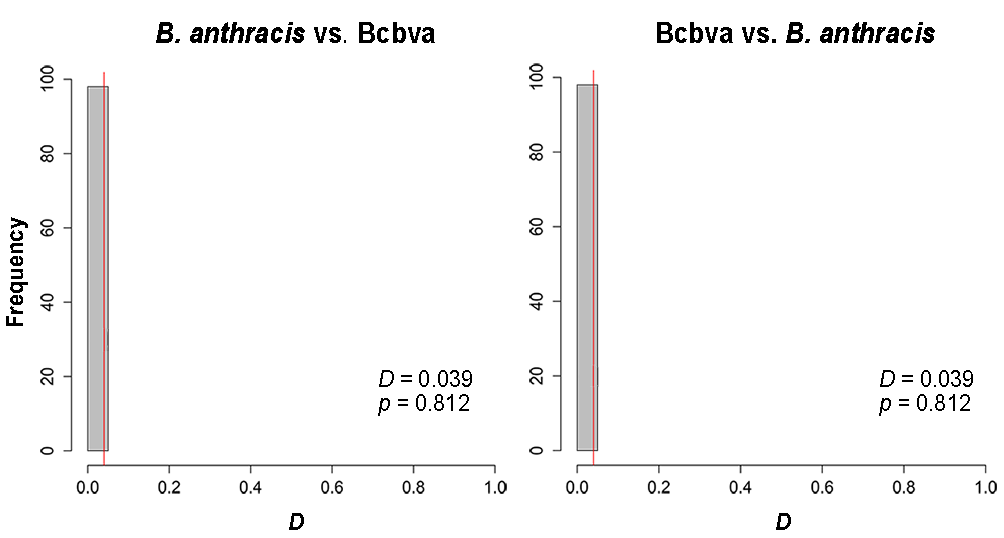

Supplement: S3 Fig — Comparison was performed calculating Schoener’s D statistic on kernel density functions on an environmental space delimited by the first two principal components of the overall set. Results are depicted for B. anthracis vs. Bcbva (left) and Bcbva vs. B. anthracis (right). (TIF) [file pntd.0008131.s006.tif]

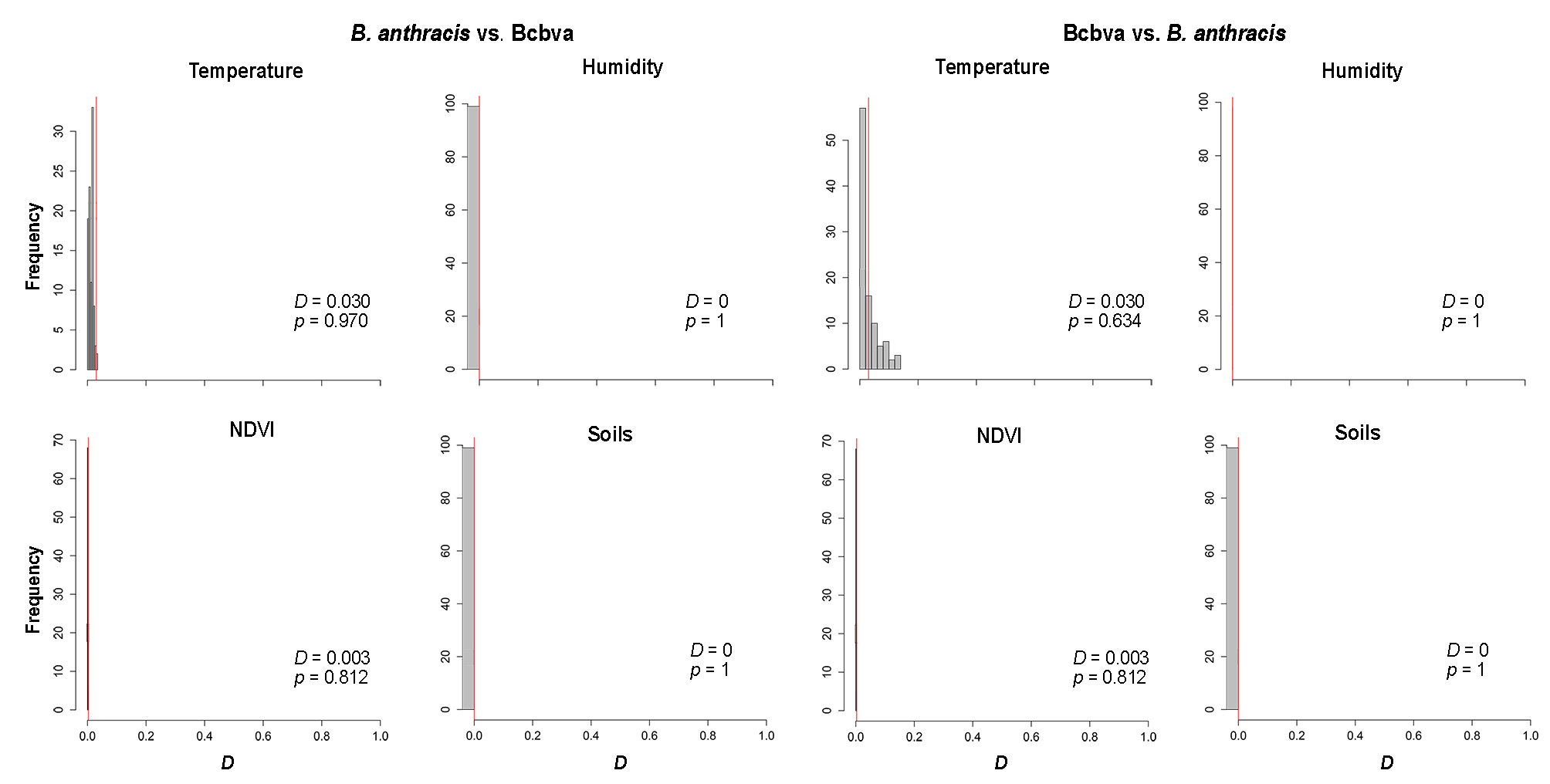

Supplement: S4 Fig — Comparison was performed calculating Schoener’s D statistic on kernel density functions on an environmental space delimited by the first two principal components of each corresponding set (i.e., temperature, humidity, NDVI, and soils). Results are depicted for B. anthracis vs. Bcbva (left) and Bcbva vs. B. anthracis (right). (TIF) [file pntd.0008131.s007.tif]

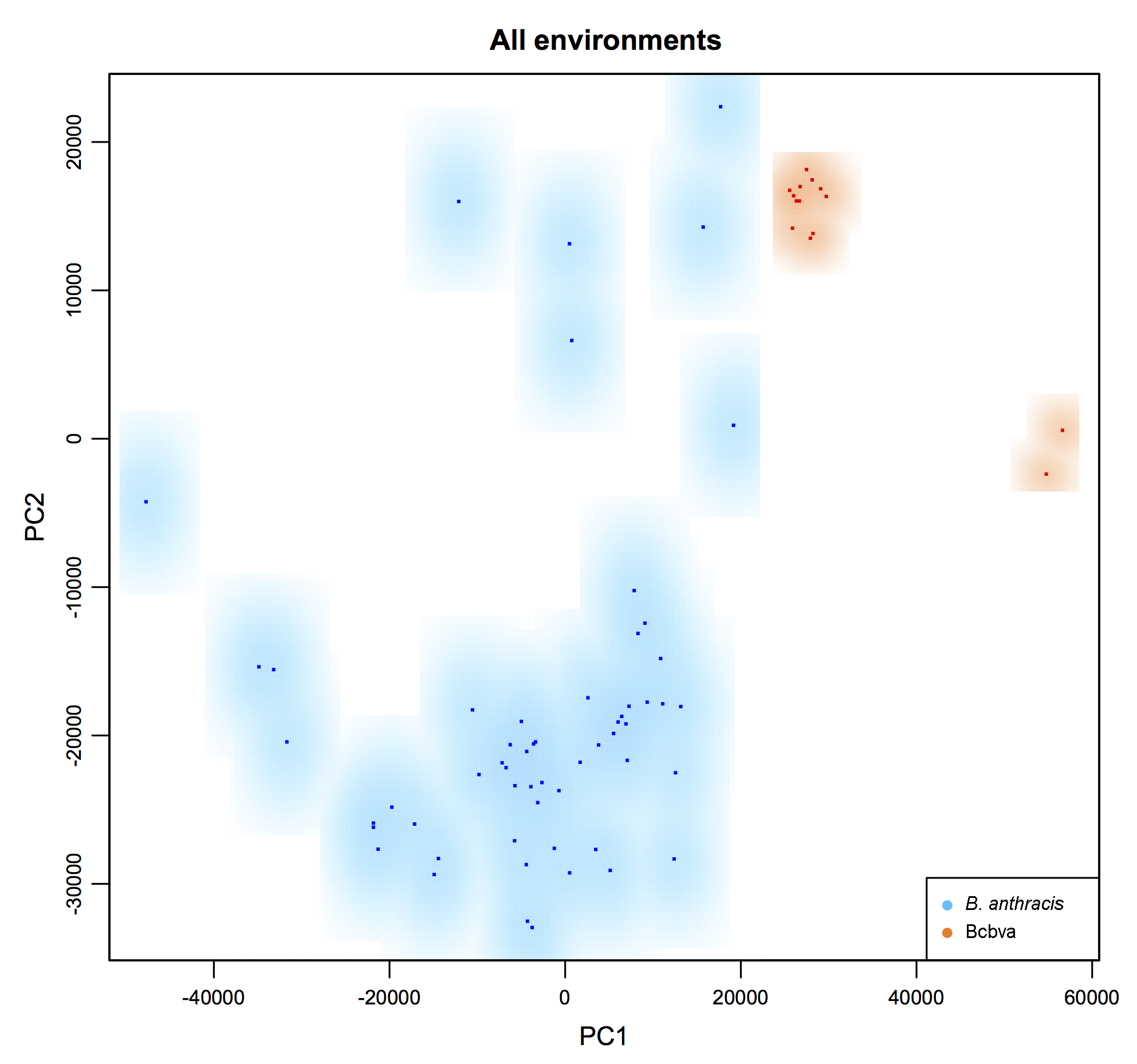

Supplement: S5 Fig — Principal components one and two (PC1 and PC2) from all the available environments (i.e., 12 variables) were used to depict an environmental space to show regions occupied by B. anthracis (blue) and Bcbva (red). Pathogens are using non-overlapping regions. (TIF) [file pntd.0008131.s008.tif]
